# Identification of a common brain network associated with lesional epilepsy

**DOI:** 10.1186/s42494-023-00138-z

**Published:** 2023-11-01

**Authors:** Di Wu, Jinghui Liu, Liankun Ren

**Affiliations:** 1 Department of Neurology, Clinical Center for Epilepsy, Xuanwu Hospital, Capital Medical University, NO.45 Changchun Street, Beijing, 100053 Xicheng District China; 2 National Center for Neurological Disorders, Beijing, 100053 China; 3 Chinese Institute for Brain Research, Beijing, 100053 China

**Keywords:** Poststroke epilepsy, Lesion network mapping, Brain network, Neuromodulation

## Abstract

Stroke is the leading cause of neurological diseases globally. Remarkably, epilepsy is a common complication of stroke, which greatly impairs the quality of life of patients and poses a significant clinical challenge. Therefore, a better understanding of the risk factors for poststroke epilepsy is crucial. A recent study published in *JAMA Neurology* studied the brain network associated with poststroke epilepsy in a group of 76 patients compared to a cohort of 625 control patients using lesion mapping techniques. The results showed that negative functional connectivity between lesion locations and regions in the basal ganglia and cerebellum confers a higher risk of developing epilepsy after stroke. The lesion network nodes associated with epilepsy were identical across different lesion types including hematomas, traumas, tumors, and tubers. Furthermore, the poststroke epilepsy brain network has potential therapeutic relevance to deep brain stimulation (DBS). In a cohort of 30 patients, the functional connectivity between anterior thalamic DBS sites and the lesion network nodes was found to correlate with seizure control after DBS. In summary, the finding provides a novel method for predicting the risk of poststroke epilepsy in patients and may guide brain stimulation treatments for epilepsy.

## Background

Stroke is the most common neurological disease globally, and the leading cause of epilepsy in the elderly. Poststroke epilepsy accounts for 9% of all epilepsy patients [1]. As a common complication of stroke, poststroke epilepsy greatly impairs the quality of patients’ life and poses a significant clinical challenge. However, currently there is no reliable method to predict the risk of developing poststroke epilepsy in clinical practice. Since epilepsy is well-recognized as a disorder of brain networks [2, 3], identifying the underlying network of poststroke epilepsy would improve clinical prediction and benefit mechanistic exploration of this common clinical manifestation.

## Main text

Using lesion mapping methods, a team from the Brigham and Women’s Hospital identified brain network maps that reveal brain regions with increased or decreased risk of poststroke epilepsy, and these findings have recently been published in *JAMA Neurology* [4]. By applying the human connectome database, the researchers used lesion mapping techniques to identify lesion locations associated with risk of poststroke epilepsy in a group of 76 patients compared to a cohort of 625 control-group patients. They found that the lesion locations related to epilepsy were more negatively connected to the substantia nigra, globus pallidus internus and cerebellum, which were referred to as “lesion network nodes”. Furthermore, they portrayed the lesion-based poststroke epilepsy brain network based on the lesion network nodes using the human connectome dataset. Finally, the network showed therapeutic relevance for deep brain stimulation (DBS), reflected by the associations between seizure control after DBS and functional connectivity of DBS stimulation site to the lesion network nodes.

This study used lesion mapping techniques including the voxel-based lesion symptom mapping technique and lesion network mapping technique to assess the specific brain regions and networks associated with poststroke epilepsy. The study enrolled 76 patients with poststroke epilepsy and 625 control patients without poststroke epilepsy. The lesions of all patients were manually segmented in high-resolution patient-specific magnetic resonance imaging scans and further linearly normalized into standard Montreal Neurological Institute stereotactic space. The traditional lesion location mapping to identify specific brain regions associated with poststroke epilepsy is conducted as follows: first, by overlapping all lesions causing epilepsy or lesions in the control group, the maximum lesion overlap was assessed on a voxel level. Then, the lesions were overlapped into priori settled regions of interest to assess if there is a specific lobe associated with epilepsy. Finally, to identify any specific voxels associated with epilepsy, univariate voxel-based lesion-symptom mapping (VLSM) and multivariate VLSM were conducted. After thorough analysis, no lesioned brain regions or individual voxels was identified to be statistically associated with epilepsy.

Next, lesion network mapping technique was applied to test whether lesions associated with poststroke epilepsy map to a specific brain network [5]. This technique has been used by a growing number of studies in recent years. The human brain connectome database derived from 1000 healthy adults (https://dataverse.harvard.edu/dataverse/GSP) was used to compute lesion network maps of personalized lesion locations [6]. Particularly, lesions negatively connected to substantia nigra, globus pallidus internus, and cerebellum (superomedial cerebellum, dentate nuclei, vermis) were found to correlate with a higher risk of poststroke epilepsy after a whole-brain voxel-based permutation test. These regions were thereby identified as lesion network nodes. Consistent results were obtained in 4 other lesion types (hematomas, traumas, tumors, and tubers). The distinct map of increased or decreased risk of poststroke epilepsy was further defined using the functional connectivity map of lesion network nodes. Visually, lesions causing poststroke epilepsy overlapped mostly within the increased risk network and vice versa. Notably, statistical analysis showed that patients whose lesions possess higher functional connectivity with the lesion network nodes were more likely to develop poststroke seizure (*P* < 0.001) compared to others. Specifically, the lesion network nodes and lesion-based poststroke epilepsy brain network have potential therapeutic implications in treating refractory epilepsy. In a cohort of 30 patients with refractory epilepsy receiving anterior thalamic DBS, those with higher functional connectivity of stimulation site to the lesion network nodes showed better improvement in seizure frequency, while DBS parameters were not correlated with seizure frequency after treatment. The findings may help guide DBS programming or refine neurosurgical modalities for treating refractory epilepsy patients.

The network derived using the lesion network mapping technique enables clinical prediction of epilepsy risk after stroke. Although the cortex is conventionally considered as the site of seizure generation, accumulating animal experimental, neuroimaging and electrophysiology evidence points to a crucial role for subcortical structures in seizure propagation, termination, and even initiation [7]. The lesion network nodes in the basal ganglia and the cerebellum highlight the crucial role of subcortical structures in epilepsy network, which may pave the way for unraveling an inhibitory network of seizures. Thus, a comprehensive understanding of the specific network may help reveal the mechanisms of refractory epilepsy and facilitate treatment of poststroke epilepsy. Furthermore, the findings mark an important step toward prediction of secondary epilepsy following a brain insult as well as improving the accuracy of predicting models to guide antiepileptogenic treatments [8].

Although the precise mechanisms underlying the therapeutic effects of DBS remain unclear, it is commonly considered that DBS targets the most influential downstream “hub” within the epileptogenic network and offers modulation within the specific network [2]. Currently, the thalamus is a hot target of neuromodulation in treating refractory epilepsy. Some subnuclei of the thalamus such as anterior thalamic nucleus (ANT), centromedian nucleus, dorsomedial nucleus, and pulvinar are emerging neuromodulation targets [9]. Of note, ANT has now been officially used to treat refractory epilepsy in clinical practice after a series of clinical trials [9]. DBS sites more connected to the basal ganglia and cerebellum are associated with better seizure control, which may explain why anterior thalamic DBS is effective across different types of focal epilepsy. Besides, selection of stimulation site and parameters of DBS is of most importance in reaching a satisfactory clinical outcome. However, this is hardly determined by the trial-and-error strategy in most clinical practice. There are no data-driven systematic methods for selecting DBS targets so far. The outcome of DBS is highly diverse across different patients [10]. The correlation of the functional connectivity between lesion network nodes and DBS sites, to the seizure frequency reduction after DBS treatment, offers a possible way to select more suitable targets and parameters. Thus, the association between lesion connectivity and epilepsy may provide insights into the improvement of clinical outcomes of refractory epilepsy after DBS treatment.

The study also had some limitations. First, it used connectivity data derived from healthy adults, so alterations in connectivity caused by stroke or epilepsy were not measured. Second, this study included patients with stroke, hematoma, trauma, tumor, and tuber related epilepsy, but the relevance of these results to other types of focal epilepsy remains to be validated. Third, the authors did not control for disease severity such as stroke severity or seizure frequency. Finally, the findings of this study were based on retrospective analysis, so further prospective testing should be done to verify if this network can be used clinically.

## Conclusions

In conclusion, the brain network for lesion-related epilepsy sheds light on prediction of lesional epilepsy and can potentially guide neuromodulation therapies for refractory epilepsy.

## Data Availability

Not applicable.

## Abbreviations

ANT: Anterior thalamic nucleus
DBS: Deep brain stimulation
VLSM: Voxel-based lesion-symptom mapping
